# Statin use during intensive care unit stay is associated with improved clinical outcomes in critically ill patients with sepsis: a cohort study

**DOI:** 10.3389/fimmu.2025.1537172

**Published:** 2025-06-06

**Authors:** Caifeng Li, Ke Zhao, Qian Ren, Lin Chen, Ying Zhang, Guolin Wang, Keliang Xie

**Affiliations:** ^1^ Department of Critical Care Medicine, Tianjin Medical University General Hospital, Tianjin, China; ^2^ Department of General Surgery, Tianjin Medical University General Hospital, Tianjin, China; ^3^ Advertising Center, Tianjin Daily, Tianjin, China; ^4^ Department of Neurosurgery, Tianjin Medical University General Hospital Airport Hospital, Tianjin, China

**Keywords:** critical illness, mortality, intensive care unit, statin, sepsis

## Abstract

**Background:**

Despite early goal-directed therapy, sepsis mortality remains high. Statins exhibit pleiotropic effects, including anti-inflammatory and antimicrobial properties, which may be beneficial during sepsis.

**Objective:**

To determine whether statins could improve the clinical outcomes in patients with sepsis.

**Methods:**

We conducted a retrospective cohort study using data from the Medical Information Mart in Intensive Care-IV (MIMIC-IV) database. Adult patients with sepsis were included in the analysis. The exposure factor of this study was statin use during the Intensive Care Unit (ICU) stay. The primary outcome was 28-day all-cause mortality. The secondary outcomes were ICU and in-hospital mortality, length of ICU stay and hospital stay, duration of mechanical ventilation (MV) and continuous renal replacement therapy (CRRT). Both propensity score matching (PSM) and stepwise regression analyses were employed to adjust for potential confounders.

**Results:**

The unmatched cohort comprised 20230 eligible patients, with 8972 patients in the statin group and 11258 in the no statin group. Propensity score matching generated balanced cohorts with 6070 patients in each group. Post-PSM analysis revealed significantly lower 28-day all-cause mortality in the statin group (14.3% [870/6070]) compared to the no statin group (23.4% [1421/6070]). Statin use was associated with decreased 28-day all-cause mortality (hazard ratio [HR], 0.56; 95% confidence interval [CI], 0.52-0.61; p < 0.001). In subgroup analysis, this beneficial effect was consistent across the different baseline characteristics of patients. Additionally, statin use was associated with decreased ICU mortality (odds ratio [OR], 0.43; 95% CI, 0.37-0.49; p < 0.001) and reduced in-hospital mortality (OR, 0.50; 95% CI, 0.45-0.57; p < 0.001). Sensitivity analysis using the unmatched cohort also showed a significant difference in 28-day all-cause mortality between the statin group and the no statin group (HR, 0.56; 95% CI, 0.52-0.61; p < 0.001).

**Conclusion:**

Statins were associated with decreased mortality in critically ill patients with sepsis. Further high-quality prospective studies are still needed to verify our findings.

## Background

Sepsis was defined as a life-threatening organ dysfunction caused by a deregulated inflammatory response to infection in the Third International Consensus Definitions released in 2016 (1). Treatment strategies for sepsis include early clinical recognition, adequate fluid resuscitation, prompt infectious source control, appropriate antibiotic therapy, and vasoactive medications as needed (2). Despite the implementation of early goal-directed therapy, the mortality rate of sepsis patients remains alarmingly high, with nearly 28% nationally (3). Sepsis is a major cause of hospitalization and mortality in the US (4, 5). To date, there is a lack of innovative adjunctive therapies to improve survival in sepsis patients (6). The complex pathophysiology of sepsis involves dysregulation of the inflammatory response, leading to an imbalance of pro- and anti-inflammatory mediators, enhanced leukocyte adhesion, inappropriate vasodilation, and impaired endothelial barrier function (7, 8). Thus, therapies aimed at modulating inflammation may hold great promise for improving clinical outcomes in sepsis (9).

Three-hydroxy-3-methylglutaryl coenzyme A (HMG-CoA) reductase inhibitors, commonly known as statins, have become one of the most widely prescribed anti-hypercholesterolemic agents (10), and play an important role in lowering morbidity and mortality associated with cardio-cerebrovascular diseases (11, 12). Besides their lipid-lowering benefits in coronary artery disease, statins exhibit lipid-independent pleiotropic effects on pro-inflammatory/anti-inflammatory cytokines, inducible nitric oxide synthase, leukocyte adhesion and rolling (13). These properties have sparked great interest in using statins as an adjunctive therapy for a variety of inflammatory disorders, including autoimmune diseases, multiple sclerosis, chronic obstructive pulmonary disease (COPD), acute respiratory distress syndrome (ARDS) and sepsis (14). In addition, studies have shown that statins have antibacterial effects, providing an additional benefit for patients with sepsis (15). Several previous studies, including real-world observational studies and meta-analyses, have demonstrated an association between statin use and improved clinical outcomes in patients with sepsis or other life-threatening inflammatory conditions (16–18). Conversely, conflicting results from other studies have shown that statin use does not always result in better health outcomes (19, 20). Therefore, it is notable that the evidence on the association between statin use and the risk of mortality and other clinical outcomes from sepsis remains inconclusive (19), partly due to their pilot design and relatively small sample sizes. While randomized controlled trials (RCTs) are considered the gold standard for generating evidence, they are difficult or impractical to conduct for their high cost, resource-intensive, time-consuming, and sometimes ethical limitations (21). Therefore, employing an advanced analytical method to mitigate the impact of measurable confounders and biases inherent in observational studies is highly recommended. The closest approximation to such a scenario is to stratify sepsis patients base on statin use during their intensive care unit (ICU) stay and to match cases to controls by propensity score on key clinical characteristics. Thus, we conducted a retrospective propensity score matched cohort study using MIMIC-IV, a large real-world database, to investigate the effect of statins on clinical outcomes in patients with sepsis.

## Materials and methods

### Data sources

We conducted a retrospective propensity score matched cohort study using the Medical Information Mart for Intensive Care-IV (MIMIC-IV), a large, freely available, de-identified, comprehensive database that includes patients admitted to the BIDMC ICUs from 2008 to 2019 (22). The database contains non-identifiable bedside health data, including demographics, vital signs, laboratory data, prescriptions, fluid balance, caregivers notes, procedural and diagnostic codes (22). A member of our team (LCF) passed the Examination of Protection of Human Research Participants and was granted access to the database (record ID: 33047414). This study was conducted and reported following the STrengthening the Reporting of OBservational Studies in Epidemiology (STROBE) statement (23).

### Study population

All consecutive patients were considered for inclusion. The inclusion criteria were as follows: (1) Diagnosed with sepsis (Sepsis-3)(1) upon hospital admission. (2) Aged 18 years or older. (3) For patients with multiple sepsis episodes and ICU stay records, only the first sepsis episode was evaluated. Patients with an ICU stay of less than 24 hours were excluded from the study.

### Medication exposure and clinical outcomes

Medication prescriptions were identified from the prescription drug file based on both generic and brand names. The medication exposure was defined simply as any statin use or no statin use during ICU stay, regardless of the statin type. The primary outcome was 28-day all-cause mortality. The secondary outcomes were ICU mortality, in-hospital mortality, length of ICU stay, length of hospital stay, duration of mechanical ventilation (MV) and continuous renal replacement therapy (CRRT). To assess the impact of statin use on clinical outcomes, eligible patients were allocated into either the statin group or the no statin group based on whether they received statins or not during their ICU stay.

### Data extraction and selection

All data were extracted from the MIMIC-IV database using Structured Query Language (SQL). The SQL script codes for data extraction were available on GitHub (https://github.com/MIT-LCP/mimic-iv). The following data were collected: demographics, including age, gender, race and body mass index (BMI); vital signs, including temperature, heart rate, respiratory rate (RR) and mean blood pressure (MBP); comorbidities, including cerebrovascular disease, congestive heart failure, chronic pulmonary disease, renal disease, severe liver disease, cancer and diabetes; severity scores, including acute physiology score III (APS III), charlson comorbidity index (CCI), glasgow coma scale (GCS), logistic organ dysfunction system (LODS), oxford acute severity of illness score (OASIS) and sequential organ failure assessment (SOFA); laboratory tests, including serum vitamin D, hemoglobin, white blood cells (WBC), platelets, creatinine, blood urea nitrogen (BUN), alanine aminotransferase (ALT), aspartate aminotransferase (AST), total bilirubin, glucose, potential of hydrogen (pH), partial pressure of oxygen (pO2), partial pressure of carbon dioxide (pCO2), partial pressure of arterial oxygen to fraction of inspired oxygen ratio (PaO2/FiO2 ratio), base excess, lactate, sodium, potassium, calcium, chloride, anion gap and international normalized ratio (INR); clinical measures, including first-day vasopressor, antibiotic lag, duration of MV and duration of CRRT. Comorbidities were assessed upon admission. Initial vital signs and clinical indices acquired within 24 hours of ICU admission were used as baseline characteristics. Variables with missing values of more than 50% were excluded from the analysis, while those with less than 50% were included. The missing rate for each variable is presented in the **Supplementary Material**: **Supplementary Table S1**, **Supplementary Figure S1**. Missing values of the included variables were imputed using the missForest method to decrease bias and avoid participant exclusion (24).

### Statistical analysis

No sample size calculation was performed as this was a retrospective exploratory study (25), and the sample size was determined by the number of patients available in the MIMIC-IV database (over 364,627 patients). Continuous variables were presented as mean (standard deviation [SD]) or median (interquartile range [IQR]) and were analyzed using either the Student’s t-test or the Mann-Whitney U-test depending on their distribution (26). Categorical variables were expressed as numbers (percentages) and compared using either the chi-square test or Fisher’s exact test (27). For the primary outcome, the Cox proportional hazards model was employed to estimate the hazard ratio (HR) and 95% confidence interval (CI). The Kaplan-Meier method and the log-rank test were used to calculate and compare the cumulative incidence of 28-day all-cause mortality. For dichotomous secondary outcomes, the logistic regression model was applied to compute the odds ratio (OR) and 95% CI. The Hodgese-Lehmann method was utilized to determine the median difference (MD) and 95% CI for continuous secondary outcomes. Multicollinearity between variables was assessed using the variance inflation factor (VIF), with VIF values of less than 5 indicating no multicollinearity (**Supplementary Material**: **Supplementary Table S2**, **Supplementary Figure S2**; **Supplementary Table S3**, **Supplementary Figure S3**). A two-tailed p < 0.05 was considered statistically significant for all analyses. All statistical analyses were performed using R software (version 4.2.3; R Foundation for Statistical Computing, Vienna, Austria).

### Propensity score matching

To address potential confounding factors and selection bias inherent in observational studies, we performed PSM following the methodological guidelines proposed by Lonjon and colleagues (28). According to a consensus statement (29), the following variables were included in the propensity score model for matching: age, gender, congestive heart failure, cerebrovascular disease, diabetes, malignant cancer, severe liver disease, APS III, CCI, heart rate, first care unit, ALT, total bilirubin, base excess, calcium, anion gap and INR. The propensity score, which represents the predicted probability of receiving statins, was calculated using baseline covariates in a logistic regression model. Patients were matched using the 1:1 nearest neighbor method without replacement and with a caliper width of 0.05. After PSM, a matched cohort of patients with comparable baseline characteristics was assembled. The covariate balance between groups was evaluated using standardized mean differences (SMD) before and after matching, with a SMD < 0.1 indicating negligible differences (30). Furthermore, Stepwise Cox regression analyses were employed to build adjusted models while adequately considering possible confounders in the matched cohort. Variables with a p-value < 0.1 in the univariate analysis were selected for further stepwise multivariate analysis. The independent variables included in the final model were age, gender, race, BMI, APS-III, CCI, LODS, OASIS, SOFA, GCS, respiratory rate, temperature, hemoglobin, WBC, creatinine, ALT, total bilirubin, pH, pCO2, lactate, calcium, potassium, anion gap, INR, antibiotic lag, first-day vasopressor and statin use (**Supplementary Material**: **Supplementary Table S4**).

### Subgroup analyses

To evaluate the impact of different variables on 28-day all-cause mortality in patients with sepsis, we conducted subgroup analyses in the matched cohort based on the following variables: age (>60 versus <=60 years), gender (female versus male), race (white, black, unknown, other), BMI (obesity, overweight, normal, underweight) and CCI (<6 versus >=6).

### Sensitivity analysis

To validate the robustness of the findings in the matched cohort, sensitivity analyses were conducted in the unmatched cohort. Stepwise Cox regression analyses were employed to identify independent prognostic factors and adjust for potential confounders. Variables with a p-value < 0.1 in the univariable analysis were entered into the multivariable analysis by stepwise selection. The independent variables incorporated into the final model were: age, gender, race, BMI, APS-III, CCI, LODS, OASIS, SOFA, GCS, respiratory rate, temperature, hemoglobin, WBC, creatinine, ALT, total bilirubin, pH, lactate, Sodium, potassium, chloride, anion gap, INR, antibiotic lag, first-day vasopressor and statin use (**Supplementary Material**: **Supplementary Table S5**).

## Result

### Patient selection

**Figure 1:**
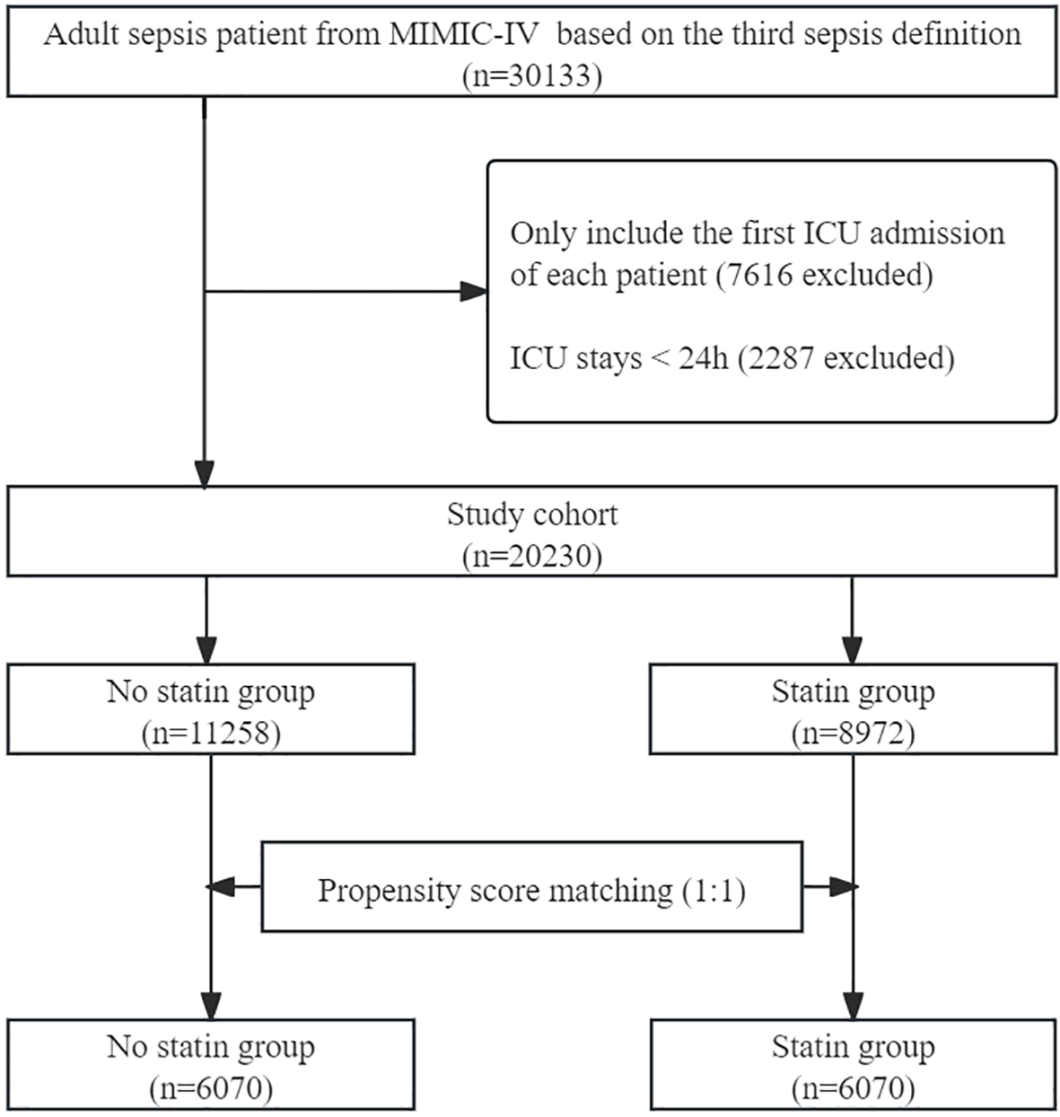
Flow chart of patient selection. MIMIC-IV, Medical Information Mart in Intensive Care-IV.

A total of 30133 adult patients with sepsis were identified from the database. After excluding ineligible records, 20230 patients were included in the unmatched cohort, with 8972 (44.34%) in statin group and 11258 (55.66%) in no statin group. After PSM, 12140 patients were included in the matched cohort, with 6070 in the statin group and 6070 in the no statin group. The process of patient selection is illustrated in **Figure 1**.

### Cohort characteristics

**Figure 2:**
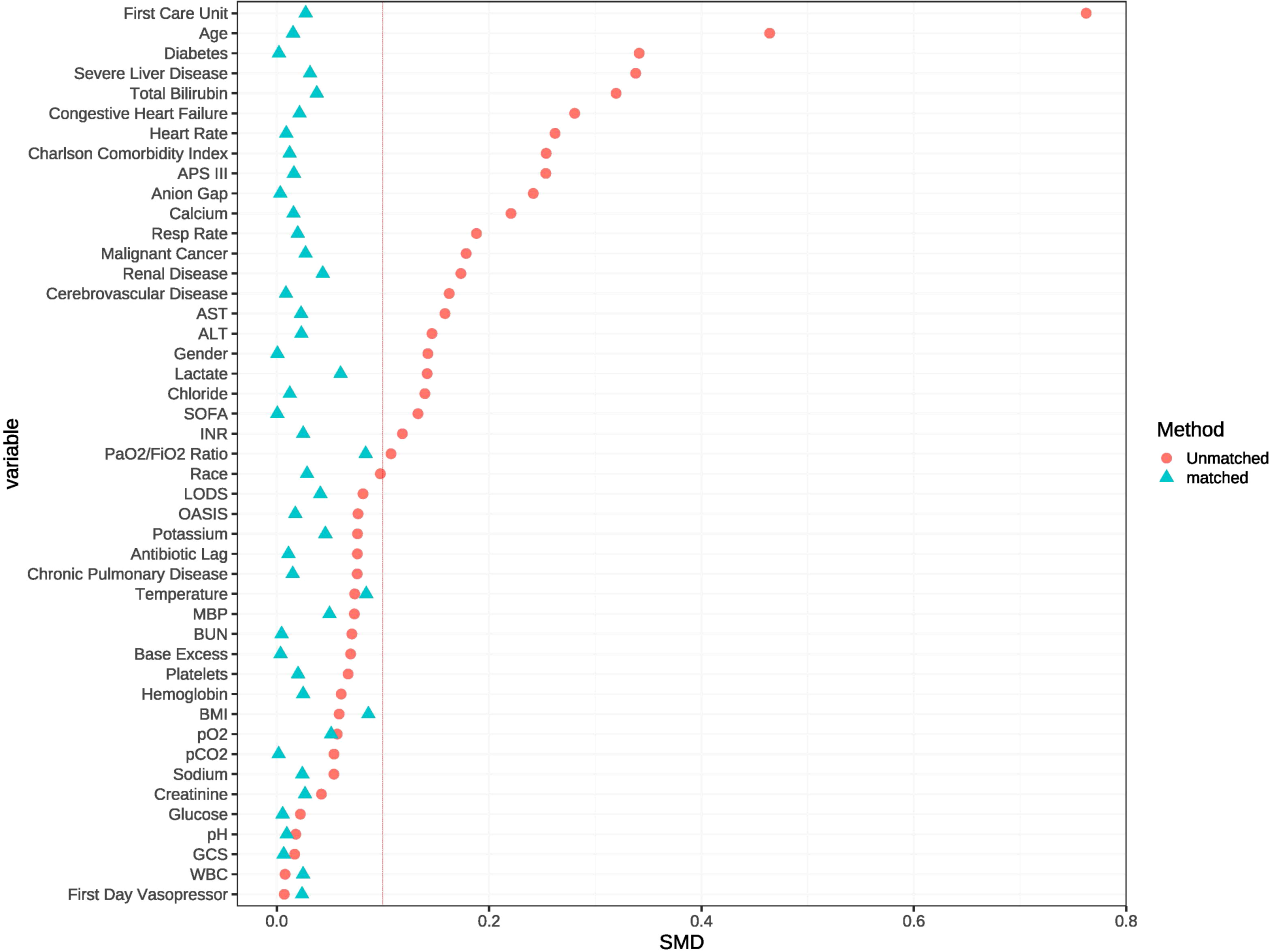
The loveplot showed SMD across covariates before and after propensity score matching.

**Figure 3:**
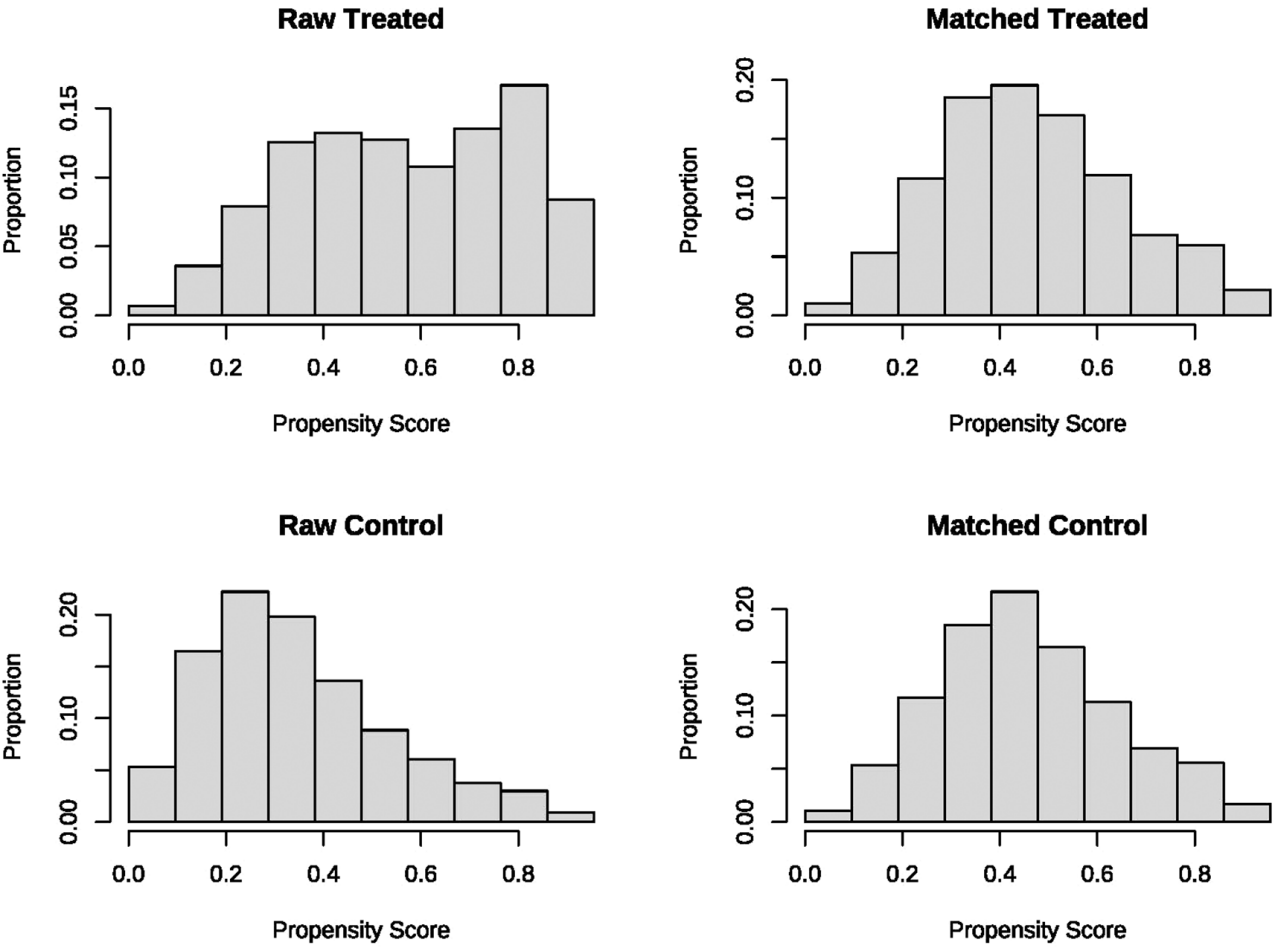
The distributional balance of propensity scores before and after propensity score matching in the two groups.

In the unmatched cohort, patients who received statin tended to be older and more likely to be male, exhibited lower APS-III and OASIS, as well as a shorter antibiotic lag. The baseline characteristics of both the unmatched and matched cohorts are shown in **Table 1**. After PSM, all variables were well-balanced in the matched cohort (SMD < 0.10) (**Figure 2**). The distribution of propensity scores of the two groups before and after matching are depicted in **Figure 3**.

**Table 1:** Baseline characteristics before and after propensity score matching.

Variable	Before propensity score matching					After propensity score matching				
	Overall	No statin	Statin	p	SMD	Overall	No statin	Statin	p	SMD
**n**	20230	11258	8972			12140	6070	6070		
Age, n (%)										
<=60	6359 (31.4)	4576 (40.6)	1783 (19.9)	<0.001	0.464	2928 (24.1)	1444 (23.8)	1484 (24.4)	0.408	0.015
>60	13871 (68.6)	6682 (59.4)	7189 (80.1)			9212 (75.9)	4626 (76.2)	4586 (75.6)		
Gender, n (%)										
Female	8458 (41.8)	5056 (44.9)	3402 (37.9)	<0.001	0.142	5148 (42.4)	2573 (42.4)	2575 (42.4)	0.985	0.001
Male	11772 (58.2)	6202 (55.1)	5570 (62.1)			6992 (57.6)	3497 (57.6)	3495 (57.6)		
Race, n (%)										
Black	1576 (7.8)	940 (8.3)	636 (7.1)	<0.001	0.098	1005 (8.3)	508 (8.4)	497 (8.2)	0.48	0.029
White	13564 (67.0)	7325 (65.1)	6239 (69.5)			8192 (67.5)	4060 (66.9)	4132 (68.1)		
Other	2074 (10.3)	1242 (11.0)	832 (9.3)			1203 (9.9)	623 (10.3)	580 (9.6)		
Unknown	3016 (14.9)	1751 (15.6)	1265 (14.1)			1740 (14.3)	879 (14.5)	861 (14.2)		
**BMI, median [IQR]**	27.69 [24.10, 32.41]	27.17 [23.52, 32.24]	28.11 [24.64, 32.51]	<0.001	0.059	28.21 [25.63, 31.18]	28.00 [25.46, 30.89]	28.39 [25.83, 31.41]	<0.001	0.086
Comorbidities, n (%)										
Congestive Heart Failure	5755 (28.4)	2572 (22.8)	3183 (35.5)	<0.001	0.281	4190 (34.5)	2064 (34.0)	2126 (35.0)	0.244	0.021
Cerebrovascular Disease	2964 (14.7)	1361 (12.1)	1603 (17.9)	<0.001	0.162	2124 (17.5)	1052 (17.3)	1072 (17.7)	0.65	0.009
Chronic Pulmonary Disease	5229 (25.8)	2744 (24.4)	2485 (27.7)	<0.001	0.076	3389 (27.9)	1674 (27.6)	1715 (28.3)	0.418	0.015
Diabetes	6059 (30.0)	2596 (23.1)	3463 (38.6)	<0.001	0.341	4064 (33.5)	2029 (33.4)	2035 (33.5)	0.923	0.002
Renal Disease	4223 (20.9)	1997 (17.7)	2226 (24.8)	<0.001	0.173	2957 (24.4)	1422 (23.4)	1535 (25.3)	0.018	0.043
Malignant Cancer	2700 (13.3)	1801 (16.0)	899 (10.0)	<0.001	0.178	1618 (13.3)	837 (13.8)	781 (12.9)	0.142	0.027
Severe Liver Disease	1407 (7.0)	1193 (10.6)	214 (2.4)	<0.001	0.338	450 (3.7)	243 (4.0)	207 (3.4)	0.093	0.031
Severity score, median [IQR]										
APS III	45.00 [34.00, 61.00]	48.00 [35.00, 64.00]	43.00 [32.00, 57.00]	<0.001	0.253	46.00 [34.00, 61.00]	46.00 [34.00, 61.00]	46.00 [35.00, 60.00]	0.856	0.016
CCI	5.00 [3.00, 7.00]	5.00 [2.00, 7.00]	5.00 [4.00, 7.00]	<0.001	0.254	5.00 [3.00, 7.00]	5.00 [3.00, 8.00]	5.00 [4.00, 7.00]	0.906	0.012
LODS	5.00 [3.00, 8.00]	5.00 [3.00, 8.00]	5.00 [3.00, 8.00]	<0.001	0.081	5.00 [3.00, 8.00]	5.00 [3.00, 8.00]	5.00 [3.00, 8.00]	0.002	0.041
OASIS	34.00 [28.00, 41.00]	35.00 [28.00, 42.00]	34.00 [28.00, 40.00]	<0.001	0.077	35.00 [29.00, 41.00]	35.00 [29.00, 41.00]	35.00 [29.00, 41.00]	0.278	0.018
SOFA	5.00 [3.00, 8.00]	5.00 [3.00, 8.00]	5.00 [3.00, 8.00]	<0.001	0.133	5.00 [3.00, 8.00]	5.00 [3.00, 8.00]	5.00 [3.00, 8.00]	0.43	0.001
GCS	15.00 [13.00, 15.00]	15.00 [13.00, 15.00]	15.00 [14.00, 15.00]	<0.001	0.017	15.00 [13.00, 15.00]	15.00 [13.00, 15.00]	15.00 [13.00, 15.00]	0.016	0.007
Vital signs, median [IQR]										
MBP	75.40 [70.01, 82.06]	75.72 [69.86, 82.82]	75.04 [70.17, 81.12]	<0.001	0.073	75.65 [70.04, 82.46]	75.48 [69.80, 82.16]	75.83 [70.24, 82.75]	0.008	0.05
Respiratory Rate	18.91 [16.69, 21.90]	19.24 [16.81, 22.47]	18.57 [16.58, 21.17]	<0.001	0.188	19.07 [16.85, 21.96]	19.00 [16.77, 22.04]	19.17 [16.94, 21.89]	0.091	0.02
Heart Rate	85.21 [75.64, 97.03]	87.52 [76.52, 99.96]	83.00 [74.85, 92.93]	<0.001	0.262	84.21 [74.64, 96.04]	84.24 [74.63, 96.28]	84.18 [74.64, 95.83]	0.749	0.009
Temperature	36.86 [36.60, 37.22]	36.89 [36.60, 37.26]	36.83 [36.59, 37.15]	<0.001	0.073	36.85 [36.60, 37.19]	36.83 [36.58, 37.17]	36.87 [36.63, 37.20]	<0.001	0.084
First Care Unit, n (%)										
CVICU	4543 (22.5)	1047 (9.3)	3496 (39.0)	<0.001	0.762	2102 (17.3)	1040 (17.1)	1062 (17.5)	0.688	0.027
MICU	4330 (21.4)	2929 (26.0)	1401 (15.6)			2628 (21.6)	1321 (21.8)	1307 (21.5)		
MICU/SICU	3697 (18.3)	2559 (22.7)	1138 (12.7)			2257 (18.6)	1156 (19.0)	1101 (18.1)		
SICU	2790 (13.8)	1872 (16.6)	918 (10.2)			1717 (14.1)	858 (14.1)	859 (14.2)		
OTHER	4870 (24.1)	2851 (25.3)	2019 (22.5)			3436 (28.3)	1695 (27.9)	1741 (28.7)		
Laboratory tests, median [IQR]										
Hemoglobin	9.70 [8.30, 11.30]	9.90 [8.30, 11.50]	9.65 [8.30, 11.10]	<0.001	0.061	9.90 [8.50, 11.40]	9.90 [8.50, 11.40]	9.90 [8.50, 11.50]	0.475	0.025
Platelets	158.00 [109.00, 221.00]	161.00 [106.00, 231.00]	155.00 [113.00, 211.00]	0.033	0.067	167.00 [117.00, 230.00]	167.00 [116.00, 234.00]	167.00 [119.00, 227.00]	0.96	0.02
WBC	14.00 [10.10, 18.90]	13.70 [9.60, 19.10]	14.30 [10.60, 18.70]	<0.001	0.008	13.80 [10.00, 18.70]	13.70 [9.80, 18.60]	14.00 [10.20, 18.70]	0.015	0.025
BUN	22.00 [15.00, 37.00]	22.00 [15.00, 38.00]	22.00 [16.00, 35.00]	0.687	0.071	24.00 [16.00, 39.00]	23.00 [16.00, 40.00]	24.00 [16.00, 39.00]	0.038	0.005
Creatinine	1.10 [0.80, 1.80]	1.10 [0.80, 1.80]	1.10 [0.80, 1.70]	0.551	0.042	1.20 [0.90, 1.80]	1.20 [0.80, 1.80]	1.20 [0.90, 1.80]	0.005	0.027
ALT	31.00 [18.00, 79.00]	34.00 [19.00, 92.00]	27.00 [16.00, 60.00]	<0.001	0.146	75.00 [24.00, 116.06]	76.13 [24.00, 117.90]	73.06 [24.00, 114.13]	0.106	0.023
AST	48.00 [27.00, 128.00]	54.00 [28.00, 147.00]	41.00 [25.00, 96.00]	<0.001	0.158	114.72 [36.00, 179.00]	115.56 [36.00, 182.03]	113.87 [36.00, 176.02]	0.162	0.023
Total Bilirubin	0.80 [0.40, 1.80]	0.90 [0.50, 2.30]	0.70 [0.40, 1.20]	<0.001	0.32	1.20 [0.60, 1.94]	1.23 [0.60, 2.00]	1.16 [0.60, 1.90]	<0.001	0.038
Glucose	131.60 [115.00, 159.00]	130.00 [110.50, 159.50]	132.67 [119.16, 157.80]	<0.001	0.022	133.00 [115.50, 165.20]	132.00 [113.60, 164.00]	134.25 [117.04, 166.70]	<0.001	0.006
pH	7.32 [7.26, 7.38]	7.33 [7.25, 7.39]	7.32 [7.27, 7.37]	0.002	0.018	7.34 [7.30, 7.37]	7.34 [7.30, 7.37]	7.34 [7.29, 7.37]	0.095	0.01
pO2	92.00 [73.00, 123.00]	91.00 [71.00, 126.00]	93.00 [75.00, 121.00]	0.005	0.057	101.00 [81.00, 126.00]	102.17 [81.00, 127.06]	100.00 [80.52, 124.00]	0.005	0.051
pCO2	46.00 [40.00, 52.00]	45.00 [38.00, 52.00]	46.00 [41.00, 52.00]	<0.001	0.054	44.74 [40.93, 49.00]	44.72 [40.73, 49.00]	44.75 [41.00, 49.00]	0.286	0.002
PaO2/FiO2 Ratio	196.67 [128.00, 281.06]	200.00 [123.33, 294.00]	194.00 [131.00, 268.00]	0.002	0.108	226.25 [168.99, 286.00]	230.00 [171.94, 291.79]	222.43 [166.67, 280.97]	<0.001	0.084
Base Excess	-3.00 [-6.00, 0.00]	-3.00 [-7.00, 0.00]	-3.00 [-5.00, 0.00]	0.818	0.07	-2.35 [-5.00, -0.56]	-2.33 [-5.00, -0.50]	-2.37 [-5.00, -0.60]	0.857	0.004
Lactate	2.30 [1.50, 3.50]	2.20 [1.40, 3.80]	2.30 [1.60, 3.30]	0.145	0.142	2.07 [1.66, 2.80]	2.09 [1.65, 2.83]	2.06 [1.66, 2.72]	0.112	0.06
Calcium	8.00 [7.50, 8.50]	7.90 [7.40, 8.40]	8.10 [7.60, 8.60]	<0.001	0.221	8.05 [7.60, 8.50]	8.00 [7.50, 8.50]	8.09 [7.60, 8.50]	0.153	0.016
Sodium	137.00 [134.00, 140.00]	137.00 [134.00, 140.00]	137.00 [135.00, 139.00]	0.002	0.054	137.00 [134.00, 140.00]	137.00 [134.00, 140.00]	137.00 [134.00, 140.00]	0.511	0.024
Potassium	4.50 [4.10, 5.00]	4.40 [4.00, 5.00]	4.50 [4.20, 5.00]	<0.001	0.076	4.50 [4.10, 5.00]	4.40 [4.10, 4.90]	4.50 [4.10, 5.00]	0.003	0.046
Chloride	103.00 [99.00, 106.00]	103.00 [98.00, 106.00]	103.00 [99.00, 107.00]	<0.001	0.14	103.00 [98.00, 106.00]	103.00 [98.00, 106.00]	103.00 [99.00, 106.00]	0.589	0.012
Anion Gap	16.00 [13.00, 19.00]	16.00 [14.00, 19.00]	15.00 [13.00, 18.00]	<0.001	0.242	16.00 [13.00, 19.00]	16.00 [13.00, 19.00]	16.00 [14.00, 19.00]	0.47	0.003
INR	1.30 [1.20, 1.60]	1.30 [1.20, 1.70]	1.30 [1.20, 1.60]	0.032	0.118	1.30 [1.20, 1.60]	1.30 [1.20, 1.60]	1.30 [1.20, 1.60]	0.44	0.025
Treatment										
Antibiotic Lag, median [IQR]	7.10 [1.55, 18.00]	7.47 [2.48, 18.06]	6.68 [0.65, 18.00]	<0.001	0.076	7.50 [2.32, 18.67]	7.46 [2.25, 18.58]	7.58 [2.35, 18.75]	0.368	0.011
First Day Vasopressor, n (%)	5944 (29.4)	3324 (29.5)	2620 (29.2)	0.627	0.007	3580 (29.5)	1757 (28.9)	1823 (30.0)	0.196	0.024

SMD, Standardized Mean Difference; IQR, Interquartile Range; APS III, Acute Physiology Score III; CCI, Charlson Comorbidity Index; LODS, Logistic Organ Dysfunction System; OASIS, Oxford Acute Severity of Illness Score; SOFA, Sequential Organ Failure Assessment Score; GCS, Glasgow Coma Scale; MBP, Mean Blood Pressure; WBC, White Blood Cell; BUN, Blood Urea Nitrogen; ALT, Alanine Aminotransferase; AST, Aspartate Aminotransferase; pH, Potential of Hydrogen; pO2, partial pressure of Oxygen; pCO2, partial pressure of Carbon Dioxide; INR, International Normalized Ratio; CVICU, Cardiac Vascular Intensive Care Unit; MICU, Medical Intensive Care Unit; MICU/SICU, Medical/Surgical Intensive Care Unit; SICU, Surgical Intensive Care Unit; PaO2/FiO2 Ratio, partial pressure of arterial oxygen to fraction of inspired oxygen ratio.Bold text represents different aspects of baseline information in **Table 1**.

### Statin regimen

In the unmatched cohort, approximately 44.34% (8972/20230) patients received statins, while in the matched cohort, approximately 50% (6070/12140) patients received statins. Various forms of statins were used during ICU stay, including atorvastatin, fluvastatin, lovastatin, pitavastatin, pravastatin, rosuvastatin, simvastatin and other statins. Clinical indications for the initiation and discontinuation of statins were not available in the database.

### Primary outcome

#### 28-day all-cause mortality

**Figure 4:**
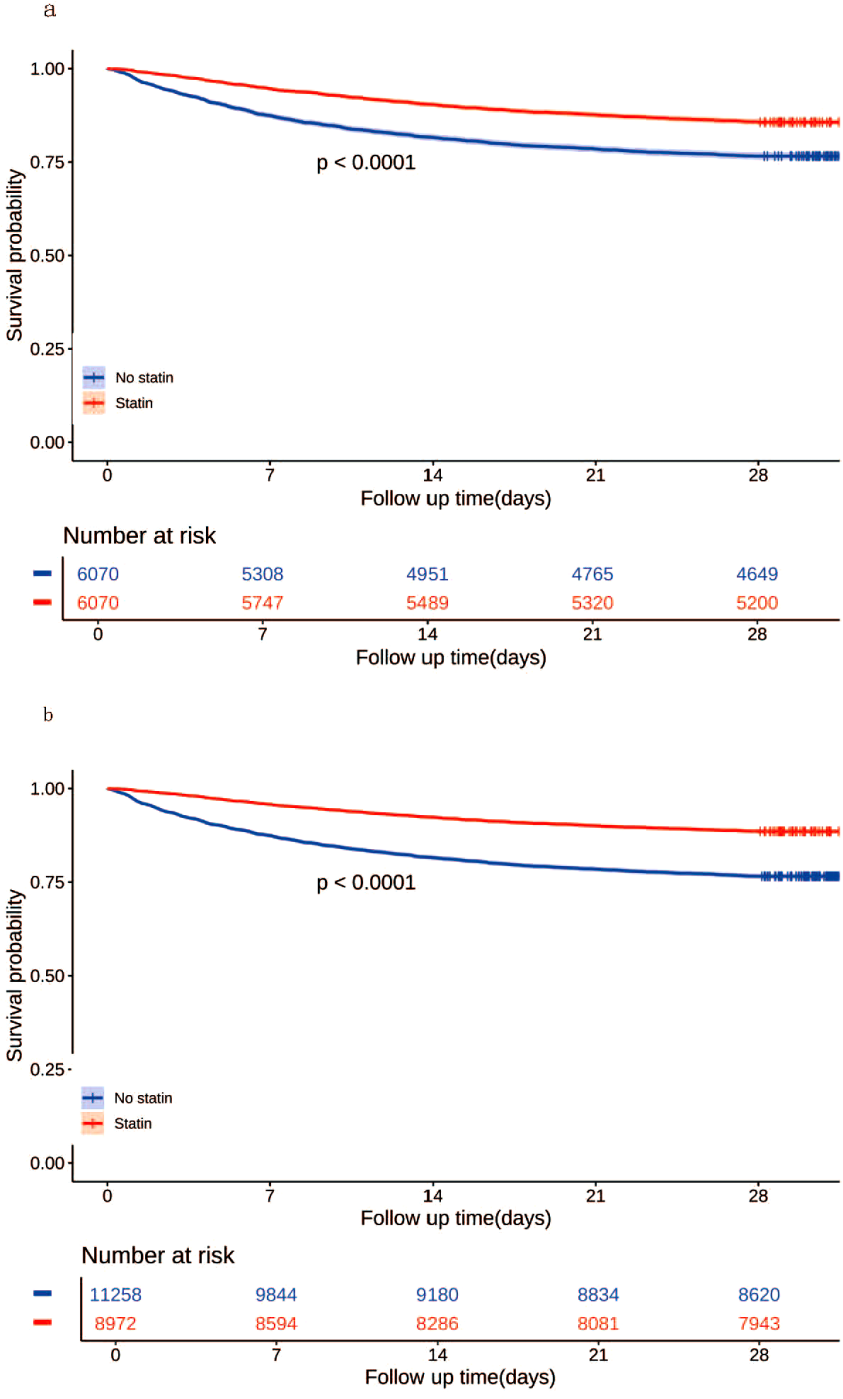
Kaplan-Meier curves for 28-day all-cause mortality according to statin use in the matched cohort **(a)** and the unmatched cohort **(b)**.

In the matched cohort, the 28-day all-cause mortality rate was 14.3% (870/6070) in the statin group and 23.4% (1421/6070) in the no statin group (p < 0.001). **Figure 4a** displays the Kaplan-Meier curve for 28-day all-cause mortality stratified by statin use in the matched cohort. Cox regression analysis indicated that statin use was associated with decreased 28-day all-cause mortality in both univariable analysis (HR, 0.57; 95% CI, 0.52-0.62; p < 0.001) and multivariable analysis (HR, 0.56; 95% CI, 0.52-0.61; p < 0.001) in the matched cohort.

#### Subgroup analyses

**Figure 5:**
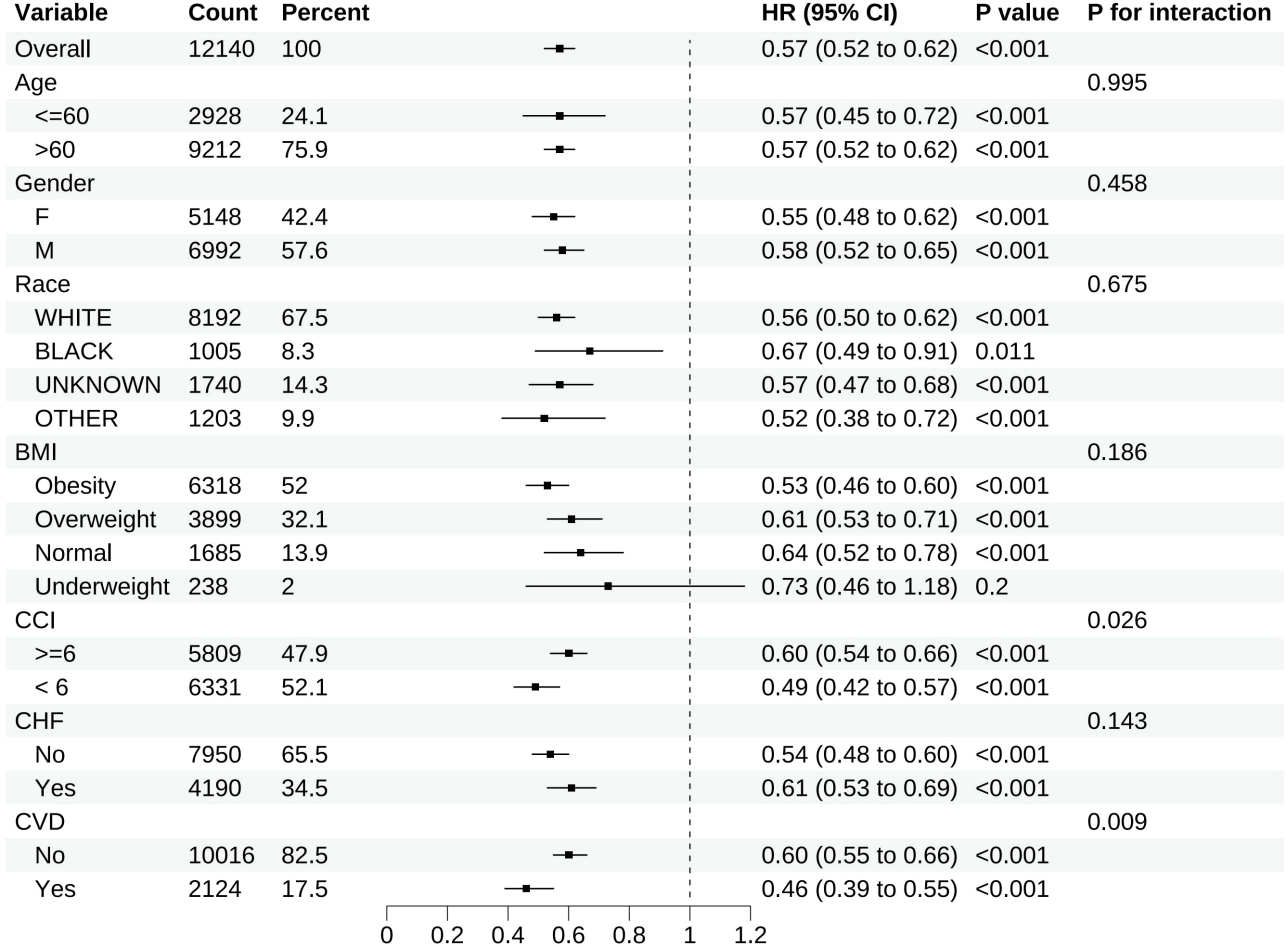
Subgroup analyses for 28-day all-cause mortality in the matched cohort.

Except for individuals categorized as underweight subgroup based on BMI, the upper limits of the 95% CIs for all other subgroups were < 1.00, indicating a reduction in 28-day all-cause mortality following in-hospital statin use regardless of baseline characteristics. Nonetheless, due to the limited sample size (n = 238) of the underweight subgroup based on BMI, this finding may be due to chance and should be interpreted with caution. The results of subgroup analyses for 28-day all-cause mortality in the matched cohort are demonstrated in **Figure 5**.

#### Sensitivity analyses

In the unmatched cohort, the 28-day all-cause mortality rate was 11.5% (1029/8972) in the statin group and 23.4% (2638/11258) in the no statin group (p < 0.001). **Figure 4b** displays the Kaplan-Meier curve for 28-day all-cause mortality stratified by statin use in the unmatched cohort. Cox regression analysis indicated that statin use was associated with decreased 28-day all-cause mortality in both univariable analysis (HR, 0.57; 95% CI, 0.52-0.62; p < 0.001) and multivariable analysis (HR, 0.56; 95% CI, 0.52-0.61; p < 0.001) in the unmatched cohort.

### Secondary outcomes

#### ICU mortality and in-hospital mortality

The ICU mortality rate was 7.4% (448/6070) in the statin group and 13.6% (826/6070) in the no statin group (p < 0.001). Logistic regression analysis showed that statin use was associated with decreased ICU mortality rate in both univariable analysis (OR, 0.51; 95% CI, 0.45-0.57; p < 0.001) and multivariable analysis (OR, 0.43; 95% CI, 0.37-0.49; p < 0.001). The in-hospital mortality rate was 11.5% (701/6070) in the statin group and 19.1% (1158/6070) in the no statin group (p < 0.001). Logistic regression analysis showed that statin use was associated with decreased in-hospital mortality rate in both univariable analysis (OR, 0.55; 95% CI, 0.50-0.61; p < 0.001) and multivariable analysis (OR, 0.50; 95% CI, 0.45-0.57; p < 0.001) (**Table 2**).

**Table 2:** The association between statin use and clinical outcomes in the matched cohort.

Outcomes	Statin (n=6070)	No statin (n=6070)	Univariable analysis		Multivariable analysis^*^	
			HR/OR/MD (95%CI)	P-value	HR/OR/MD (95% CI)	P-value
Primary outcome						
28-day mortality^@^, n (%)	870 (14.3)	1421 (23.4)	0.57 (0.52-0.62)	<0.001	0.56 (0.52-0.61)	<0.001
Secondary outcomes						
					
ICU mortality^$^, n (%)	448 (7.4)	826 (13.6)	0.51 (0.45-0.57)	<0.001	0.43 (0.37-0.49)	<0.001
In-hospital mortality^$^, n (%)	701 (11.5)	1158 (19.1)	0.55 (0.50-0.61)	<0.001	0.50 (0.45-0.57)	<0.001
Length of ICU stay^¶^ (days), median [IQR]	3.58 [1.93, 7.79]	3.06 [1.86, 6.02]	0.34 (0.25-0.43)	<0.001		
Length of hospital stay^¶^ (days), median [IQR]	9.86 [5.94, 17.36]	8.32 [5.11, 14.51]	1.44 (1.22-1.67)	<0.001		
Ventilation Duration^¶^ (days), median [IQR]	41.47 [14.15, 133.00]	36.79 [13.00, 103.97]	3.00 (1.47-4.65)	<0.001		
CRRT Duration^¶^ (days), median [IQR]	106.74 [44.56, 202.87]	68.00 [22.55, 147.86]	26 (10.00-43.38)	<0.001		

CI, confidence interval; HR, hazard ratio; IQR, interquartile range; MD, median difference; OR, odds ratio; CRRT, continuous renal replacement therapy. ^*^ Adjusted for age, gender, race, BMI, APS-III, CCI, LODS, OASIS, SOFA, GCS, respiratory rate, temperature, hemoglobin, WBC, creatinine, ALT, total bilirubin, pH, pCO2, lactate, calcium, potassium, anion gap, INR, antibiotic lag and first-day vasopressor. **^@^** HR with 95% CI was calculated using the Cox proportional hazards model. **^$^** OR with 95% CI was calculated using the logistic regression model. ^¶^MD with 95% CI was calculated using the Hodges-Lehmann estimator. Bold text represents different aspects of outcomes in **Table 2**.

#### Duration of MV and CRRT

The median duration of MV was 41.47 hours (IQR, 14.15-133.00) in the statin group, while in the no-statin group, it was 36.79 hours (IQR, 13.00-103.97). Similarly, the median duration of CRRT was 106.74 hours (IQR, 44.56-202.87) in the statin group, while in the no-statin group, it was 68.00 hours (IQR, 22.55-147.86). Statin use was associated with prolonged duration of MV (MD, 3.00 hours; 95% CI, 1.47-4.65; p < 0.001) and CRRT (MD, 26.00 hours; 95% CI, 10.00-43.38; p < 0.001), but not with shortened duration of MV and CRRT (**Table 2**).

#### Length of ICU stay and hospital stay

The median length of ICU stay was 3.58 days (IQR, 1.93-7.79) in the statin group and 3.06 days (IQR, 1.86-6.02) in the no statin group. Similarly, the median length of hospital stay was 9.86 days (IQR, 5.94-17.36) in the statin group and 8.32 days (IQR, 5.11-14.51) in the no statin group. Statin use was associated with prolonged length of ICU stay (MD, 0.34 days; 95% CI, 0.25-0.43; p < 0.001) and hospital stay (MD, 1.44 days; 95% CI, 1.22-1.67; p < 0.001), but not with shortened length of ICU stay and hospital stay (**Table 2**).

## Discussion

In a large real-world clinical setting, we conducted a retrospective propensity score matched cohort study to evaluate the association between statin use and mortality among 20230 patients with sepsis. We found that statin users exhibited decreased 28-day all-cause mortality in both the matched and unmatched cohorts. Our subgroup analyses by BMI category revealed statistically significant protective effects of statins on sepsis in normal weight (HR, 0.64; 95% CI, 0.52-0.78; p < 0.001), overweight (HR, 0.61; 95% CI, 0.53-0.71; p < 0.001), and obese patients (HR, 0.53; 95% CI, 0.46-0.60; p < 0.001). While the point estimate for underweight patients showed a similar trend (HR, 0.73; 95% CI, 0.46-1.18; p = 0.2), this subgroup did not reach statistical significance, likely due to limited sample size (n=238, 2%) rather than a true biological difference. The result was consistent and stable in sensitivity analyses, indicating the robustness of our finding. Notably, statin therapy demonstrated associations with reduced ICU mortality and in-hospital mortality, prolonged ICU and hospital stay, and increased duration of MV and CRRT. These paradoxical findings likely reflect competing risk dynamics, wherein the mortality benefit permits extended survival of critically ill patients requiring prolonged intensive care and organ support (**Table 2**). This is supported by evidence from multiple studies demonstrating that statin use is associated with reduced mortality in critically ill patients, including those with sepsis (31). The prolonged duration of mechanical ventilation and CRRT should therefore be interpreted as a reflection of the complex interplay between disease severity, comorbidities, and the potential benefits of statin therapy, rather than as a negative outcome. In conclusion, our study suggests that statin use during the ICU stay may exert a protective effect in patients with sepsis.

### Relation with previous evidence

While randomized controlled trials are widely regarded as the gold standard of evidence-based medicine, conducting prospective randomized controlled trials to assess the effect of statin use on sepsis prognosis is challenging due to the large number of patients required to achieve a sufficient cohort of patients who actually develop sepsis. We believe that the best alternative to a prospective randomized controlled trial is exactly what we have done: identify a cohort, follow them over time, even if not concurrently, and match cases to controls by propensity matching on important clinical characteristics.

To account for the selection bias and unmeasured confounders inherent in observational studies, we employed the PSM approach (32) to ensure that all patients were pseudo-randomized to the treatment and control groups as in a typical RCT. PSM enables the generation of an unbiased average treatment effect of statin on clinical outcomes among patients with sepsis admitted to the ICU. PSM allows simultaneous modeling of the propensity for unbiased group allocation and modeling of the outcomes using multivariate regression adjustment, thereby obtaining double robust and unbiased estimates of the average treatment effect of statins (32).

After adjusting for various biases inherent in observational studies using PSM, we observed a beneficial effect of statin use on the outcome of sepsis, which is contrary to the findings of several RCTs. Though the methodological differences between RCTs and observational studies are frequently cited as the primary source of such discrepancy, our study employed a pseudo-randomized quasi-experimental approach that successfully adjusted for selection biases and supported the results of most observational studies (16, 33, 34).

Our findings challenge the previous assertion made by Majumda that a healthy user effect explains why observational studies demonstrated the beneficial effects of statins on sepsis patients (35). Because the use of the PSM approach allows individuals to be assigned randomly to different groups, thus eliminating the possibility of a healthy user effect (20).

There are several possible reasons why most RCTs failed to detect a beneficial effect of statins in patients with sepsis. A comprehensive review of these RCTs revealed that sepsis diagnoses were often underreported, and many trials could not provide additional data upon request, increasing the risk that a non-representative sample of statin-treated patients was enrolled and assessed for sepsis outcomes (20). It is noteworthy that the PSM approach used in this study is not inherently superior to large-scale RCTs with complete data reporting, but it helps mitigate biases in observational studies and address noncompliance issues in RCTs.

### Possible explanations for our findings

Subgroup analyses revealed consistent beneficial effects of statin therapy in sepsis patients irrespective of pre-existing cerebrovascular diseases and chronic heart failure. Notably, while the plaque-stabilizing properties of statins constitute the primary mechanism underlying their cardiovascular protective effects, this observed sepsis-associated mortality reduction in both subgroups suggest potential pleiotropic mechanisms independent of atherosclerotic plaque modulation.

The pleiotropic effects of statins have been well documented in the literature. However, despite this, the underlying mechanism by which statins confer benefit in sepsis remains unclear (36). Potential explanations for this beneficial effect include: First, statins may attenuate the severity of sepsis through their anti-inflammatory, immunomodulatory, antioxidative, and antithrombotic effects (37–40). In animal models of sepsis, statins have been demonstrated to inhibit the elevation of inflammatory mediators (41, 42), resulting in improved survival rates (43, 44). Previous clinical studies have shown that statins may exert potential antioxidant properties in models of sepsis, which could help mitigate tissue damage and organ dysfunction (45). Statins have been reported to inhibit the expression of toll-like receptors (TLR) 4 and 2 on monocytes in human endotoxemia models, leading to a decrease in inflammatory cytokine production (45). Statins may interfere with transcription factors such as nuclear factor kappaB (NF-kappaB) and activation protein-1 (AP-1), which could result in a reduction in the synthesis of proinflammatory cytokines, including interleukin-1 (IL-1) and IL-6 (46). Similarly, an association between statin treatment and reduced levels of tumor necrosis factor (TNF) and IL-6 has been observed in patients experiencing acute bacterial infections (47). Statins have been demonstrated to inhibit adhesion molecules in both neutrophils/monocytes and endothelial cells, resulting in a decreased migration of polynuclear neutrophils into tissues (48–50). Statins may assist in restoring the balance between endothelial nitric oxide synthase (eNOS) and inducible nitric oxide synthase (iNOS), which is disrupted in sepsis (51). By substantially boosting eNOS expression while downregulating iNOS, statins have the potential to prevent or reverse sepsis-related endothelial dysfunction (51). Furthermore, statins may play a crucial role in mitigating the negative effects of sepsis on the coagulation system by inhibiting the expression of tissue factor and plasminogen activator inhibitor-1, improving protein C function (52), lowering prothrombin fragment levels, and significantly upregulating the expression of thrombomodulin (53, 54). Second, Statin use was associated with a lower risk of bacterial infection. Statins may have direct antimicrobial properties (37, 55), as the enzymes in the mevalonate pathway, which are potentially modified by statin therapy, are also involved in the development of Gram-positive bacterial infections (55). It is noteworthy that statins may also exhibit antifungal properties due to the similarities between the ergosterol biosynthetic pathway in fungi and the cholesterol synthesis in humans, implying a direct effect on Candida species (56, 57). The immunomodulatory, antioxidative, anti-inflammatory, antithrombotic, and direct antimicrobial effects of statins may account for the beneficial effects against sepsis observed in our study.

### Strength and limitation

The main strength of this study lies in the utilization of the PSM analytical approach, which allows for the generation of doubly robust unbiased estimates of the average treatment effects of statins in patients with sepsis. However, this study also has several limitations. First, the observational design inherently precludes definitive causal inferences, as unmeasured confounding factors may influence the observed associations despite our rigorous propensity score matching and multivariable adjustment approaches. Second, the study may be subject to potential residual confounders that are not recorded in the MIMIC-IV database. Although PSM is a robust method for addressing multiple baseline differences between groups, variables included in this study are confined to relevant variables available in the MIMIC-IV database, potentially introducing bias from unmeasured confounders. Third, the study did not identify the specific effects of individual statins on sepsis. In this study, the exposure was simply defined as either the use of any statin or no statin during the ICU stay. Previous studies have demonstrated that simvastatin, atorvastatin and rosuvastatin exhibit antibacterial properties, while other statins do not (15, 58). As a result, studies conducted without distinguishing the effects of different statins are prone to underestimate their effects, and future studies should be conducted to compare the clinical outcomes associated with individual statins. Fourth, the impact of prior statin use on clinical outcomes was not investigated. The study focused only on statin use during the ICU stay. However, pretreatment with simvastatin has been demonstrated to improve sepsis survival in mouse models by preserving cardiac function, lowering circulatory inflammatory cytokines, decreasing neutrophil migration to the lung, and enhancing T-cell function (42, 43, 59). Therefore, studies conducted without considering the impact of prior statin exposure may overestimate the beneficial effects of statins.

## Conclusion

From a large, population-based cohort study, we found an association between statin use and reduced sepsis-related mortality. Given the wide use of statins for the prevention of cardiovascular disease, it is likely that their use in this population has also conferred benefits in combating infections and sepsis.

## Data Availability

The datasets presented in this study can be found in online repositories. The names of the repository/repositories and accession number(s) can be found below: Publicly available datasets were used in this study. This data is available here: https://physionet.org/content/mimiciv/2.0/.

## Supplementary material

Supplementary Figure 1 Percentage of missing data of each variable.

Supplementary Figure 2 Variance inflation factor of each variable in the matched cohort.

Supplementary Figure 3 Variance inflation factor of each variable in the unmatched cohort.

Supplementary Table 1 Percentage of missing data of each variable.

Supplementary Table 2 Variance inflation factor of each variable in the matched cohort.

Supplementary Table 3 Variance inflation factor of each variable in the unmatched cohort.

Supplementary Table 4 Cox regression model for 28-day all-cause mortality using stepwise selection in the matched cohort.

Supplementary Table 5 Cox regression model for 28-day all-cause mortality using stepwise selection in the unmatched cohort.
