# Supplementary figures and images for: Statin use during intensive care unit stay is associated with improved clinical outcomes in critically ill patients with sepsis: a cohort study
Source: Front Immunol. 2025 Jun 6;16:1537172. doi: 10.3389/fimmu.2025.1537172 (PMC12179067; doi:10.3389/fimmu.2025.1537172)

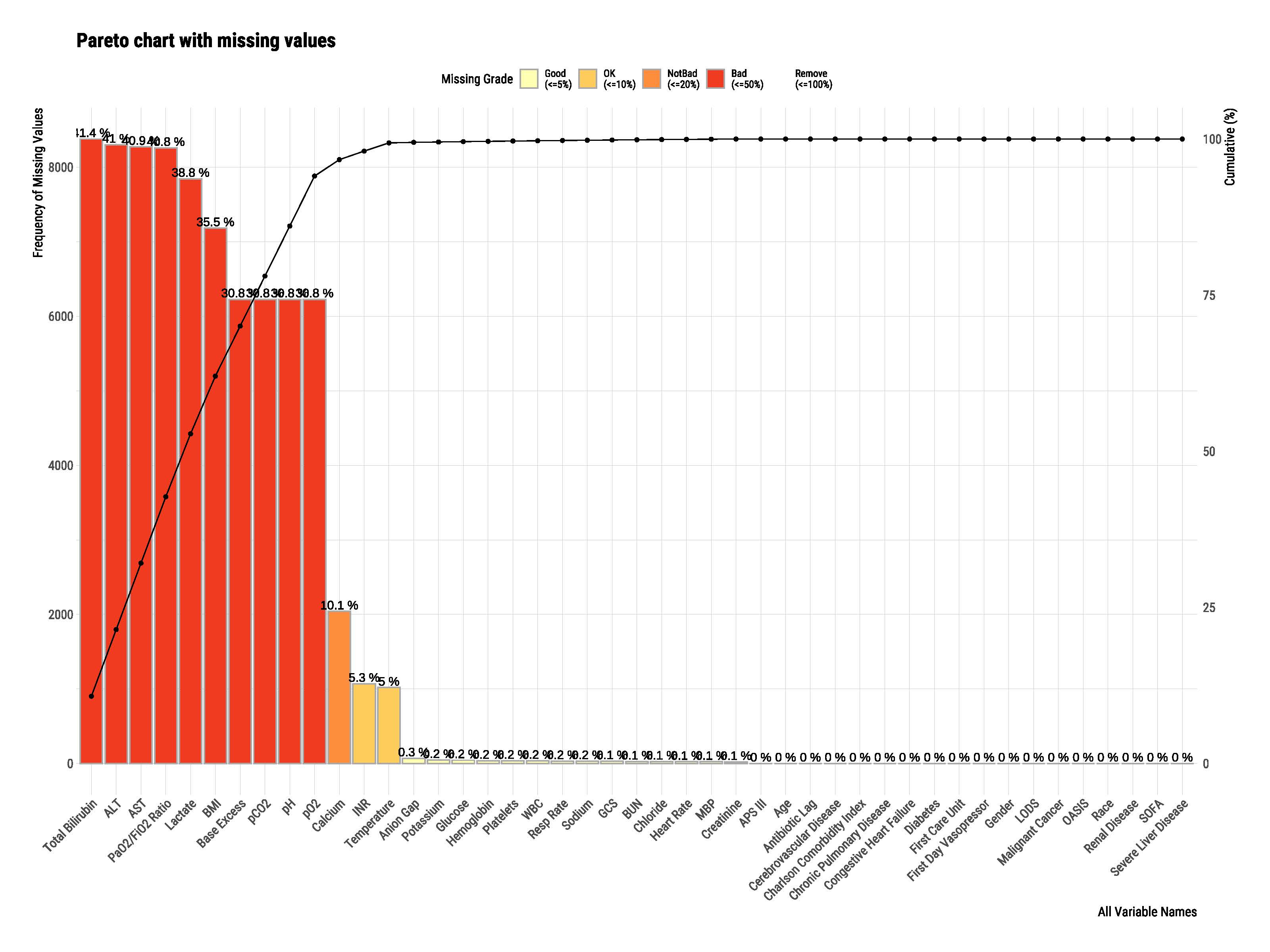

Supplement: Supplementary Figure 1 — Percentage of missing data of each variable. [file Image1.jpeg]

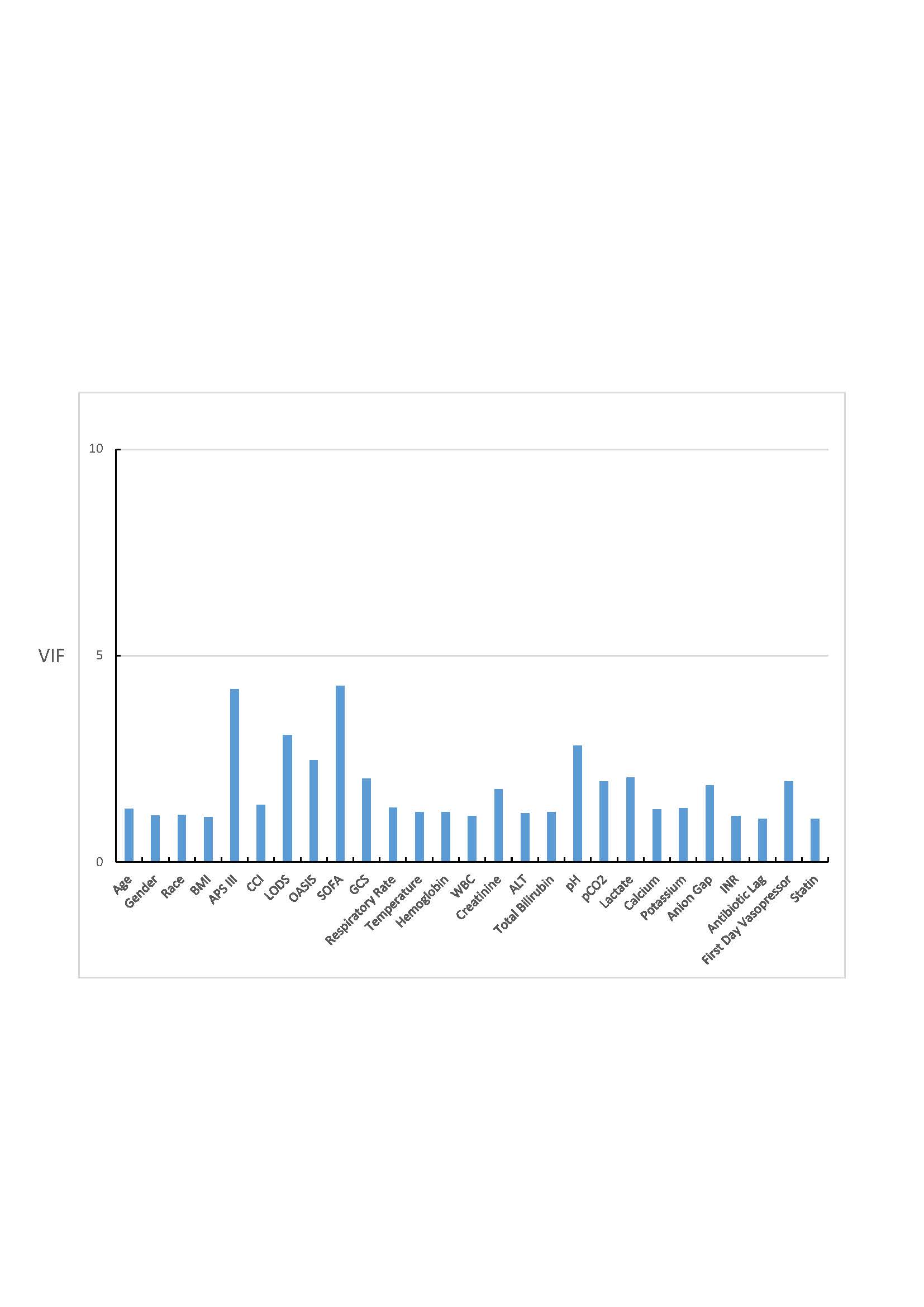

Supplement: Supplementary Figure 2 — Variance inflation factor of each variable in the matched cohort. [file Image2.jpeg]

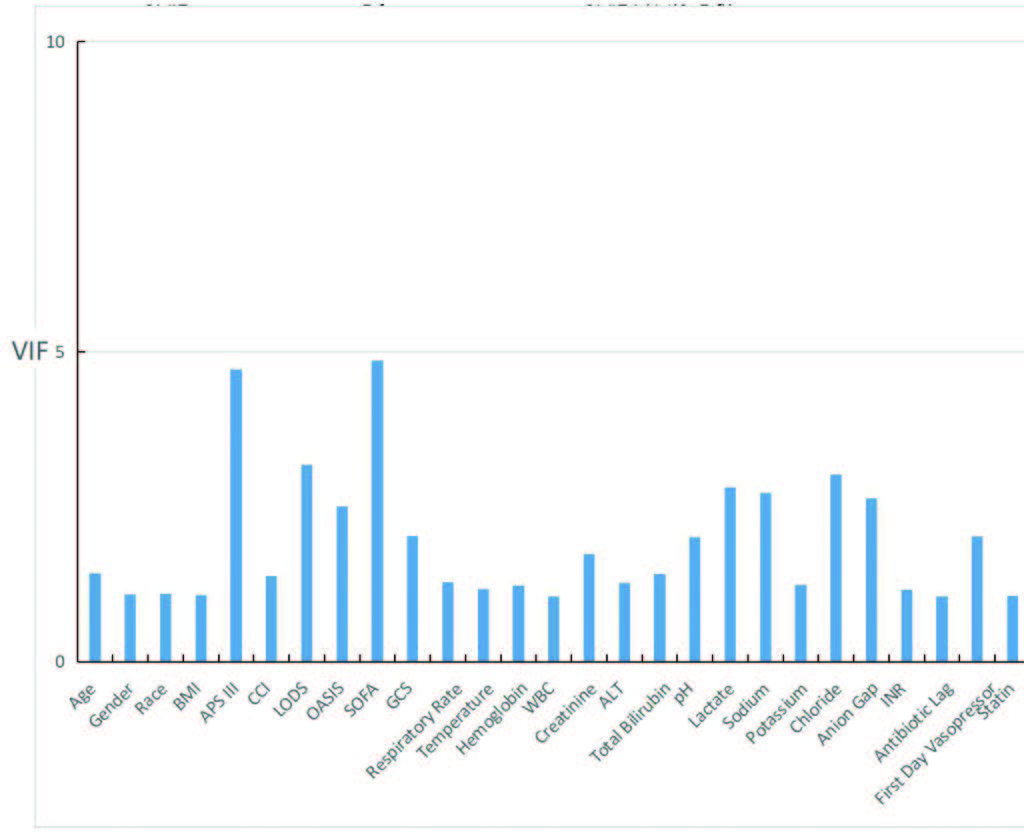

Supplement: Supplementary Figure 3 — Variance inflation factor of each variable in the unmatched cohort. [file Image3.jpeg]
