# Supplementary material for: Statin use during intensive care unit stay is associated with improved clinical outcomes in critically ill patients with sepsis: a cohort study
Source: Front Immunol. 2025 Jun 6;16:1537172. doi: 10.3389/fimmu.2025.1537172 (PMC12179067; doi:10.3389/fimmu.2025.1537172)
Supplement: Supplementary Table 1 — Percentage of missing data of each variable. [file Table1.docx]

Table S1. Percentage of missing data of each variable.

| **Variables** | **Missing count** | **Missing percent** |
| --- | --- | --- |
| Age | 0 | 0.00 |
| Gender | 0 | 0.00 |
| Race | 0 | 0.00 |
| BMI | 7181 | 35.50 |
| Congestive Heart Failure | 0 | 0.00 |
| Cerebrovascular Disease | 0 | 0.00 |
| Chronic Pulmonary Disease | 0 | 0.00 |
| Diabetes | 0 | 0.00 |
| Renal Disease | 0 | 0.00 |
| Malignant Cancer | 0 | 0.00 |
| Severe Liver Disease | 0 | 0.00 |
| APS III | 0 | 0.00 |
| CCI | 0 | 0.00 |
| LODS | 0 | 0.00 |
| OASIS | 0 | 0.00 |
| SOFA | 0 | 0.00 |
| GCS | 30 | 0.15 |
| MBP | 23 | 0.11 |
| Respiratory Rate | 33 | 0.16 |
| Heart Rate | 23 | 0.11 |
| Temperature | 1018 | 5.03 |
| First Care Unit | 0 | 0.00 |
| Hemoglobin | 37 | 0.18 |
| Platelets | 36 | 0.18 |
| WBC | 35 | 0.17 |
| BUN | 27 | 0.13 |
| Creatinine | 21 | 0.10 |
| ALT | 8302 | 41.04 |
| AST | 8272 | 40.89 |
| Total Bilirubin | 8377 | 41.41 |
| Glucose | 44 | 0.22 |
| pH | 6227 | 30.78 |
| pO2 | 6227 | 30.78 |
| pCO2 | 6227 | 30.78 |
| PaO2/FiO2 Ratio | 8258 | 40.82 |
| Base Excess | 6227 | 30.78 |
| Lactate | 7844 | 38.77 |
| Calcium | 2040 | 10.08 |
| Sodium | 32 | 0.16 |
| Potassium | 46 | 0.23 |
| Chloride | 27 | 0.13 |
| Anion Gap | 69 | 0.34 |
| INR | 1069 | 5.28 |
| Antibiotic Lag | 0 | 0.00 |
| First Day Vasopressor | 0 | 0.00 |
