# Supplementary material for: Statin use during intensive care unit stay is associated with improved clinical outcomes in critically ill patients with sepsis: a cohort study
Source: Front Immunol. 2025 Jun 6;16:1537172. doi: 10.3389/fimmu.2025.1537172 (PMC12179067; doi:10.3389/fimmu.2025.1537172)
Supplement: Supplementary Table 2 — Variance inflation factor of each variable in the matched cohort. [file Table2.docx]

Table S2. Variance inflation factor of each variable in the matched cohort.

| Variables | Variance inflation factor(VIF) |
| --- | --- |
| Age | 1.286872 |
| Gender | 1.12392 |
| Race | 1.143493 |
| BMI | 1.084483 |
| APS III | 4.189523 |
| CCI | 1.383224 |
| LODS | 3.080521 |
| OASIS | 2.470581 |
| SOFA | 4.275505 |
| GCS | 2.018476 |
| Respiratory Rate | 1.319852 |
| Temperature | 1.216769 |
| Hemoglobin | 1.217137 |
| WBC | 1.108651 |
| Creatinine | 1.767487 |
| ALT | 1.175471 |
| Total Bilirubin | 1.204824 |
| pH | 2.824708 |
| pCO2 | 1.948529 |
| Lactate | 2.046018 |
| Calcium | 1.271426 |
| Potassium | 1.309724 |
| Anion Gap | 1.855197 |
| INR | 1.11565 |
| Antibiotic Lag | 1.044808 |
| First Day Vasopressor | 1.947842 |
| Statin use | 1.040532 |
