# Supplementary material for: Statin use during intensive care unit stay is associated with improved clinical outcomes in critically ill patients with sepsis: a cohort study
Source: Front Immunol. 2025 Jun 6;16:1537172. doi: 10.3389/fimmu.2025.1537172 (PMC12179067; doi:10.3389/fimmu.2025.1537172)
Supplement: Supplementary Table 3 — Variance inflation factor of each variable in the unmatched cohort. [file Table3.docx]

Table S3. Variance inflation factor of each variable in the unmatched cohort.

| Variables | Variance inflation factor (VIF) |
| --- | --- |
| Age | 1.423811 |
| Gender | 1.083703 |
| Race | 1.1006 |
| BMI | 1.077217 |
| APS III | 4.715205 |
| CCI | 1.380448 |
| LODS | 3.181087 |
| OASIS | 2.508949 |
| SOFA | 4.86268 |
| GCS | 2.028986 |
| Respiratory Rate | 1.287715 |
| Temperature | 1.176945 |
| Hemoglobin | 1.22647 |
| WBC | 1.054204 |
| Creatinine | 1.734316 |
| ALT | 1.27373 |
| Total Bilirubin | 1.419881 |
| pH | 2.006596 |
| Lactate | 2.809379 |
| Sodium | 2.728655 |
| Potassium | 1.238277 |
| Chloride | 3.017008 |
| Anion Gap | 2.634368 |
| INR | 1.163561 |
| Antibiotic Lag | 1.050873 |
| First Day Vasopressor | 2.024446 |
| Statin use | 1.069373 |
