# Supplementary material for: Statin use during intensive care unit stay is associated with improved clinical outcomes in critically ill patients with sepsis: a cohort study
Source: Front Immunol. 2025 Jun 6;16:1537172. doi: 10.3389/fimmu.2025.1537172 (PMC12179067; doi:10.3389/fimmu.2025.1537172)
Supplement: Supplementary Table 4 — Cox regression model for 28-day all-cause mortality using stepwise selection in the matched cohort. [file Table4.pdf]

Dependent: Surv(Time, Status)		all	HR (univariable)	HR (multivariable)	HR (final)
Age	<=60	2928 (24.1%)			
	>60	9212 (75.9%)	2.44 (2.15-2.76, p<.001)	1.23 (1.07-1.42, p=.003)	1.24 (1.08-1.42, p=.002)
Gender	F	5148 (42.4%)			
	M	6992 (57.6%)	0.90 (0.83-0.98, p=.015)	0.90 (0.82-0.98, p=.016)	0.90 (0.82-0.98, p=.014)
Race	BLACK	1005 (8.3%)			
	OTHER	1203 (9.9%)	0.84 (0.68-1.04, p=.114)	1.05 (0.84-1.30, p=.670)	1.06 (0.85-1.32, p=.599)
	UNKNOWN	1740 (14.3%)	1.90 (1.60-2.27, p<.001)	2.17 (1.81-2.60, p<.001)	2.19 (1.83-2.62, p<.001)
	WHITE	8192 (67.5%)	1.08 (0.92-1.27, p=.345)	1.29 (1.09-1.52, p=.003)	1.31 (1.11-1.54, p=.001)
BMI	Mean ± SD	28.9 ± 6.3	0.97 (0.97-0.98, p<.001)	0.98 (0.97-0.98, p<.001)	0.98 (0.97-0.98, p<.001)
APS.III	Mean ± SD	49.4 ± 20.7	1.03 (1.02-1.03, p<.001)	1.01 (1.00-1.01, p<.001)	1.01 (1.00-1.01, p<.001)
Charlson.Comorbidity.Index	Mean ± SD	5.5 ± 2.9	1.20 (1.19-1.22, p<.001)	1.16 (1.14-1.18, p<.001)	1.16 (1.14-1.18, p<.001)
LODS	Mean ± SD	5.9 ± 3.3	1.22 (1.21-1.24, p<.001)	1.15 (1.12-1.17, p<.001)	1.15 (1.13-1.17, p<.001)
OASIS	Mean ± SD	35.3 ± 9.2	1.07 (1.07-1.08, p<.001)	1.02 (1.01-1.03, p<.001)	1.02 (1.01-1.03, p<.001)
SOFA	Mean ± SD	5.7 ± 3.3	1.15 (1.13-1.16, p<.001)	0.97 (0.95-0.99, p=.010)	0.97 (0.95-0.99, p=.006)
GCS	Mean ± SD	13.2 ± 3.1	0.96 (0.95-0.97, p<.001)	1.03 (1.02-1.05, p<.001)	1.03 (1.02-1.05, p<.001)
MBP	Mean ± SD	76.9 ± 10.1	0.99 (0.98-0.99, p<.001)	1.00 (0.99-1.00, p=.626)	
Resp.Rate	Mean ± SD	19.7 ± 3.9	1.09 (1.08-1.10, p<.001)	1.05 (1.04-1.06, p<.001)	1.05 (1.04-1.06, p<.001)
Heart.Rate	Mean ± SD	85.9 ± 15.9	1.01 (1.01-1.01, p<.001)	1.00 (1.00-1.00, p=.902)	
Temperature	Mean ± SD	36.9 ± 0.6	0.65 (0.61-0.70, p<.001)	0.79 (0.74-0.85, p<.001)	0.79 (0.74-0.84, p<.001)
Hemoglobin	Mean ± SD	10.0 ± 2.1	0.97 (0.95-0.99, p=.003)	1.06 (1.03-1.08, p<.001)	1.05 (1.03-1.08, p<.001)
Platelets	Mean ± SD	184.4 ± 102.0	1.00 (1.00-1.00, p=.130)		
WBC	Mean ± SD	15.6 ± 11.8	1.01 (1.01-1.01, p<.001)	1.00 (1.00-1.01, p=.035)	1.00 (1.00-1.01, p=.034)
BUN	Mean ± SD	31.9 ± 24.5	1.01 (1.01-1.01, p<.001)	1.00 (1.00-1.00, p=.854)	
Creatinine	Mean ± SD	1.7 ± 1.7	1.07 (1.05-1.09, p<.001)	0.91 (0.88-0.95, p<.001)	0.92 (0.88-0.95, p<.001)
ALT	Mean ± SD	132.4 ± 392.8	1.00 (1.00-1.00, p<.001)	1.00 (1.00-1.00, p=.532)	1.00 (1.00-1.00, p=.167)

n=12140, events=2291, Likelihood ratio test=2654.65 on 37 df(p<.001)
cluster=subclass

Dependent: Surv(Time, Status)		all	HR (univariable)	HR (multivariable)	HR (final)
AST	Mean ± SD	220.7 ± 787.6	1.00 (1.00-1.00, p<.001)	1.00 (1.00-1.00, p=.828)	
Total.Bilirubin	Mean ± SD	1.7 ± 2.6	1.03 (1.02-1.04, p<.001)	1.02 (1.01-1.03, p=.001)	1.02 (1.01-1.03, p=.001)
Glucose	Mean ± SD	291.6 ± 6911.9	1.00 (1.00-1.00, p=.188)		
pH	Mean ± SD	7.3 ± 0.1	0.15 (0.10-0.24, p<.001)	6.31 (1.30-30.57, p=.022)	5.99 (3.08-11.66, p<.001)
pO2	Mean ± SD	110.9 ± 53.1	1.00 (1.00-1.00, p<.001)	1.00 (1.00-1.00, p=.862)	
pCO2	Mean ± SD	46.2 ± 11.4	0.99 (0.99-1.00, p=.012)	1.00 (1.00-1.01, p=.310)	1.00 (1.00-1.01, p=.049)
PaO2.FiO2.Ratio	Mean ± SD	230.8 ± 95.8	1.00 (1.00-1.00, p<.001)	1.00 (1.00-1.00, p=.937)	
Base.Excess	Mean ± SD	-3.0 ± 4.7	0.95 (0.94-0.95, p<.001)	1.00 (0.97-1.02, p=.834)	
Lactate	Mean ± SD	2.6 ± 1.9	1.15 (1.14-1.17, p<.001)	1.04 (1.01-1.06, p=.005)	1.04 (1.01-1.06, p=.004)
Calcium	Mean ± SD	8.0 ± 0.8	0.92 (0.87-0.96, p<.001)	1.06 (1.01-1.12, p=.019)	1.06 (1.01-1.12, p=.020)
Sodium	Mean ± SD	136.8 ± 5.4	1.01 (1.00-1.01, p=.185)		
Potassium	Mean ± SD	4.6 ± 0.9	1.24 (1.19-1.29, p<.001)	1.05 (1.00-1.10, p=.064)	1.05 (1.00-1.10, p=.053)
Chloride	Mean ± SD	102.1 ± 6.6	0.98 (0.98-0.99, p<.001)	1.00 (0.99-1.00, p=.308)	
Anion.Gap	Mean ± SD	16.8 ± 5.0	1.07 (1.07-1.08, p<.001)	1.03 (1.01-1.04, p<.001)	1.03 (1.02-1.04, p<.001)
INR	Mean ± SD	1.6 ± 1.1	1.15 (1.13-1.18, p<.001)	1.03 (1.00-1.06, p=.041)	1.03 (1.00-1.06, p=.038)
Antibiotic.Lag	Mean ± SD	14.2 ± 17.7	1.00 (1.00-1.01, p=.002)	1.00 (1.00-1.00, p=.094)	1.00 (1.00-1.00, p=.089)
First.Day.Vasopressor	No	8560 (70.5%)			
	Yes	3580 (29.5%)	1.86 (1.71-2.02, p<.001)	1.13 (1.00-1.27, p=.047)	1.14 (1.01-1.28, p=.032)
Statin	No	6070 (50.0%)			
	Yes	6070 (50.0%)	0.57 (0.52-0.62, p<.001)	0.56 (0.52-0.61, p<.001)	0.56 (0.52-0.61, p<.001)

n=12140, events=2291, Likelihood ratio test=2654.65 on 37 df(p<.001)
cluster=subclass
