# Supplementary material for: Statin use during intensive care unit stay is associated with improved clinical outcomes in critically ill patients with sepsis: a cohort study
Source: Front Immunol. 2025 Jun 6;16:1537172. doi: 10.3389/fimmu.2025.1537172 (PMC12179067; doi:10.3389/fimmu.2025.1537172)
Supplement: Supplementary Table 5 — Cox regression model for 28-day all-cause mortality using stepwise selection in the unmatched cohort. [file Table5.pdf]

Dependent: Surv(Time, Status)		all	HR (univariable)	HR (multivariable)	HR (final)
Age	<=60	6359 (31.4%)			
	>60	13871 (68.6%)	1.58 (1.46-1.71, p<.001)	1.11 (1.01-1.22, p=.033)	1.11 (1.01-1.22, p=.024)
Gender	F	8458 (41.8%)			
	M	11772 (58.2%)	0.86 (0.81-0.92, p<.001)	0.90 (0.84-0.96, p=.002)	0.90 (0.84-0.96, p=.002)
Race	BLACK	1576 (7.8%)			
	OTHER	2074 (10.3%)	0.88 (0.75-1.04, p=.142)	1.07 (0.91-1.26, p=.423)	1.07 (0.91-1.26, p=.419)
	UNKNOWN	3016 (14.9%)	1.83 (1.59-2.10, p<.001)	2.05 (1.78-2.36, p<.001)	2.05 (1.78-2.36, p<.001)
	WHITE	13564 (67.0%)	0.97 (0.86-1.11, p=.678)	1.19 (1.05-1.36, p=.008)	1.19 (1.05-1.36, p=.007)
BMI	Mean ± SD	28.9 ± 6.3	0.98 (0.98-0.99, p<.001)	0.98 (0.98-0.99, p<.001)	0.98 (0.98-0.99, p<.001)
APS.III	Mean ± SD	49.4 ± 21.9	1.03 (1.03-1.03, p<.001)	1.01 (1.01-1.02, p<.001)	1.01 (1.01-1.02, p<.001)
Charlson.Comorbidity.Index	Mean ± SD	5.1 ± 2.9	1.19 (1.18-1.20, p<.001)	1.16 (1.14-1.17, p<.001)	1.16 (1.15-1.17, p<.001)
LODS	Mean ± SD	5.8 ± 3.4	1.24 (1.23-1.25, p<.001)	1.14 (1.12-1.16, p<.001)	1.14 (1.12-1.16, p<.001)
OASIS	Mean ± SD	35.0 ± 9.3	1.08 (1.08-1.08, p<.001)	1.01 (1.01-1.02, p<.001)	1.01 (1.01-1.02, p<.001)
SOFA	Mean ± SD	5.9 ± 3.5	1.17 (1.17-1.18, p<.001)	0.97 (0.95-0.99, p=.001)	0.97 (0.96-0.99, p=.001)
GCS	Mean ± SD	13.2 ± 3.2	0.95 (0.94-0.96, p<.001)	1.05 (1.03-1.06, p<.001)	1.05 (1.03-1.06, p<.001)
MBP	Mean ± SD	76.7 ± 10.1	0.99 (0.98-0.99, p<.001)	1.00 (1.00-1.00, p=.650)	
Resp.Rate	Mean ± SD	19.6 ± 4.0	1.10 (1.09-1.11, p<.001)	1.04 (1.04-1.05, p<.001)	1.05 (1.04-1.05, p<.001)
Heart.Rate	Mean ± SD	86.8 ± 16.0	1.01 (1.01-1.02, p<.001)	1.00 (1.00-1.00, p=.128)	1.00 (1.00-1.00, p=.118)
Temperature	Mean ± SD	36.9 ± 0.6	0.64 (0.61-0.67, p<.001)	0.79 (0.76-0.84, p<.001)	0.79 (0.75-0.83, p<.001)
Hemoglobin	Mean ± SD	9.9 ± 2.1	0.96 (0.95-0.98, p<.001)	1.06 (1.04-1.07, p<.001)	1.06 (1.04-1.07, p<.001)
Platelets	Mean ± SD	176.2 ± 101.6	1.00 (1.00-1.00, p=.850)		
WBC	Mean ± SD	15.8 ± 12.2	1.01 (1.01-1.01, p<.001)	1.00 (1.00-1.00, p=.005)	1.00 (1.00-1.00, p=.005)
BUN	Mean ± SD	30.3 ± 24.0	1.01 (1.01-1.01, p<.001)	1.00 (1.00-1.00, p=.301)	
Creatinine	Mean ± SD	1.7 ± 1.7	1.10 (1.08-1.11, p<.001)	0.91 (0.88-0.93, p<.001)	0.91 (0.89-0.94, p<.001)
ALT	Mean ± SD	168.9 ± 758.6	1.00 (1.00-1.00, p<.001)	1.00 (1.00-1.00, p=.008)	1.00 (1.00-1.00, p<.001)

n=20230, events=3667, Likelihood ratio test=5042.64 on 38 df(p<.001)

Dependent: Surv(Time, Status)		all	HR (univariable)	HR (multivariable)	HR (final)
AST	Mean ± SD	281.2 ± 1141.8	1.00 (1.00-1.00, p<.001)	1.00 (1.00-1.00, p=.807)	
Total.Bilirubin	Mean ± SD	2.1 ± 3.8	1.05 (1.04-1.05, p<.001)	1.02 (1.02-1.03, p<.001)	1.02 (1.02-1.03, p<.001)
Glucose	Mean ± SD	350.2 ± 9576.2	1.00 (1.00-1.00, p=.269)		
pH	Mean ± SD	7.3 ± 0.1	0.08 (0.06-0.12, p<.001)	5.26 (1.63-16.93, p=.005)	3.07 (2.06-4.59, p<.001)
pO2	Mean ± SD	110.6 ± 52.4	1.00 (0.99-1.00, p<.001)	1.00 (1.00-1.00, p=.203)	
pCO2	Mean ± SD	46.3 ± 11.5	0.99 (0.99-1.00, p=.001)	1.00 (1.00-1.01, p=.335)	
PaO2.FiO2.Ratio	Mean ± SD	228.5 ± 95.7	1.00 (1.00-1.00, p<.001)	1.00 (1.00-1.00, p=.335)	
Base.Excess	Mean ± SD	-3.2 ± 4.8	0.93 (0.93-0.94, p<.001)	0.99 (0.97-1.01, p=.339)	
Lactate	Mean ± SD	2.7 ± 2.1	1.16 (1.15-1.17, p<.001)	1.02 (1.00-1.04, p=.016)	1.02 (1.01-1.04, p=.008)
Calcium	Mean ± SD	8.0 ± 0.8	0.86 (0.83-0.90, p<.001)	1.02 (0.98-1.06, p=.351)	
Sodium	Mean ± SD	136.7 ± 5.3	0.99 (0.99-1.00, p=.006)	1.02 (1.01-1.03, p<.001)	1.02 (1.02-1.03, p<.001)
Potassium	Mean ± SD	4.6 ± 0.9	1.20 (1.18-1.23, p<.001)	1.07 (1.03-1.11, p<.001)	1.08 (1.04-1.12, p<.001)
Chloride	Mean ± SD	102.3 ± 6.6	0.97 (0.96-0.97, p<.001)	0.98 (0.97-0.99, p<.001)	0.98 (0.97-0.98, p<.001)
Anion.Gap	Mean ± SD	16.6 ± 5.2	1.07 (1.07-1.08, p<.001)	1.02 (1.01-1.03, p<.001)	1.02 (1.01-1.03, p<.001)
INR	Mean ± SD	1.6 ± 1.1	1.17 (1.15-1.18, p<.001)	1.04 (1.02-1.06, p<.001)	1.04 (1.02-1.06, p<.001)
Antibiotic.Lag	Mean ± SD	13.6 ± 17.4	1.01 (1.00-1.01, p<.001)	1.00 (1.00-1.00, p=.006)	1.00 (1.00-1.00, p=.006)
First.Day.Vasopressor	No	14286 (70.6%)			
	Yes	5944 (29.4%)	2.05 (1.92-2.19, p<.001)	1.11 (1.01-1.22, p=.027)	1.10 (1.01-1.21, p=.036)
Statin	No	11258 (55.7%)			
	Yes	8972 (44.3%)	0.45 (0.42-0.48, p<.001)	0.48 (0.44-0.52, p<.001)	0.48 (0.45-0.52, p<.001)

n=20230, events=3667, Likelihood ratio test=5042.64 on 38 df(p<.001)
